# Hindbrain V2a Neurons Pattern Rhythmic Activity of Motor Neurons in a Reticulospinal Coculture

**DOI:** 10.3389/fnins.2019.01077

**Published:** 2019-10-17

**Authors:** Adele Bubnys, Hagar Kandel, Lee Ming Kao, Donald Pfaff, Inna Tabansky

**Affiliations:** ^1^Laboratory of Neurobiology and Behavior, The Rockefeller University, New York, NY, United States; ^2^Feinstein Institute for Medical Research, Manhasset, NY, United States

**Keywords:** Chx10 neurons, Hb9 neurons, multielectrode arrays, reticulospinal neurons, cell type specific activity, hindbrain V2a neurons

## Abstract

As the capacity to isolate distinct neuronal cell types has advanced over the past several decades, new two- and three-dimensional *in vitro* models of the interactions between different brain regions have expanded our understanding of human neurobiology and the origins of disease. These cultures develop distinctive patterns of activity, but the extent that these patterns are determined by the molecular identity of individual cell types versus the specific pattern of network connectivity is unclear. To address the question of how individual cell types interact *in vitro*, we developed a simplified culture using two excitatory neuronal subtypes known to participate in the *in vivo* reticulospinal circuit: HB9^+^ spinal motor neurons and Chx10^+^ hindbrain V2a neurons. Here, we report the emergence of cell type-specific patterns of activity in culture; on their own, Chx10^+^ neurons developed regular, synchronized bursts of activity that recruited neurons across the entire culture, whereas HB9^+^ neuron activity consisted of an irregular pattern. When these two subtypes were cocultured, HB9^+^ neurons developed synchronized network bursts that were precisely correlated with Chx10^+^ neuron activity, thereby recreating an aspect of Chx10^+^ neurons’ role in driving motor activity. These bursts were dependent on AMPA receptors. Our results demonstrate that the molecular classification of the neurons comprising *in vitro* networks is a crucial determinant of their activity. It is therefore possible to improve both the reproducibility and the applicability of *in vitro* neurobiological and disease models by carefully controlling the constituent mixtures of neuronal subtypes.

## Introduction

The nervous system is composed of thousands of types of neurons with distinct molecular and anatomical properties (Tabansky et al., 2015). These neurons are organized into circuits with specific patterns of activity that enable an organism to perform adaptive behaviors. The early life experiences of an organism are essential for reinforcing adaptive patterns of neuronal activity (Hensch, 2005; Fagiolini et al., 2009; Leslie and Nedivi, 2011). But in order to ensure rapid and effective development, the behavior of these circuits and the molecular properties of their constituent neurons must be at least partially genetically encoded (Shen and Scheiffele, 2010). This genetic encoding is exploited by models of neuronal disease grown in cell culture dishes, which are not functionally constrained by the rest of the nervous system.

To derive clinically relevant findings, most *in vitro* models of neurological disease seek to incorporate as realistic a mixture of cells from the modeled region as possible. Such models hold therapeutic promise, as some have already been used to identify small molecule candidates for drug development (Yang et al., 2013; Wainger et al., 2015; Ahfeldt et al., 2017). Models of CNS regions have also been shown to develop complex patterns of activity similar to their *in vivo* counterparts (Trujillo et al., 2018). Primary tissue taken from different areas of the brain and cultured on multi-electrode arrays (MEAs) develops regular bursts of spiking activity with tissue-specific differences in the shape of spike waveforms and the timing and structure of bursts (Dauth et al., 2016; Soscia et al., 2017; Sarkar et al., 2018). When the different tissue types were co-cultured on a single array they developed correlated activity, suggesting that they could communicate with each other.

However, it is unclear whether these observations of bursts and changes in activity are an emergent property of the mixture of cell types in these cultures or governed by the presence of specific neuronal cell types (Chiappalone et al., 2006; Maheswaranathan et al., 2012; Lonardoni et al., 2017). To appreciate how network activity emerges in such cultures and how it is determined by the properties of individual neuronal cell types, it is important to study each cell type in isolation prior to combining them into a more complex system.

To address this question, we created a simple system from two neuronal subtypes known to participate at different levels of the locomotor control system, motor neurons and reticulospinal hindbrain neurons. Motor neurons relay patterned input from spinal cord central pattern generator circuits to skeletal muscles to initiate behavior (Binder et al., 1996). Reticulospinal neurons are components of a prominent behavioral circuit that relays rhythmic locomotor drive from the brainstem to the spinal cord (Peterson, 1979; Garcia-Rill and Skinner, 1987a, b; Le Ray et al., 2011; Kiehn, 2016).

Of the subtypes of reticulospinal neurons, we specifically focused on V2a excitatory interneurons, identified by expression of the transcription factor Ceh-10 Homeodomain-Containing Homolog (Chx10, also known as Visual System Homeobox 2, or Vsx2), which are found in the spinal cord and medullary reticular formation. Chx10^+^ V2a neurons within the hindbrain reticular formation play a role in regulating hindlimb locomotion (Bretzner and Brownstone, 2013; Bouvier et al., 2015; Cregg et al., 2019) and respiratory rhythm (Crone et al., 2009, 2012) and have functional connectivity to the mesencephalic locomotor region and pre-Bötzinger complex.

In rodents, reticulospinal neurons are critical for setting the timing and gait of locomotion without altering the left-right and flexor-extensor alternation important for the correct expression of gait (Hägglund et al., 2010; Rybak et al., 2015). Glutamatergic reticulospinal neurons are known to form polysynaptic contacts with motor neurons by way of commissural interneurons that participate in the rhythm generating component of the spinal central pattern generator (Noga et al., 2003; Jankowska et al., 2005; Szokol et al., 2011; Lemieux and Bretzner, 2019). The specific class of interneuron that hindbrain Chx10^+^ neurons contact has not yet been identified, but these neurons have been shown to be involved in transmitting locomotor stop and turn signals that are relayed via premotor networks within the spinal cord, rather than by direct synapses with motor neurons (Bouvier et al., 2015; Cregg et al., 2019).

By contrast, in the zebrafish and *Xenopus* tadpole, hindbrain Chx10^+^ neurons directly contact spinal motor neurons and provide patterned excitatory input that is critical for sensory-evoked swimming (Soffe et al., 2009; Kimura et al., 2013; Li and Soffe, 2019). A similar patterning role can be seen among V2a neurons of the rodent spinal cord, which form an important component of the pattern generating circuitry responsible for left-right alternation (Crone et al., 2008; Dougherty and Kiehn, 2010; Hayashi et al., 2018; Song et al., 2018). When cultured as a purified population *in vitro*, these spinal V2a neurons developed spontaneous coordinated bursting activity consistent with their role in rhythm generation (Sternfeld et al., 2017).

In our simplified reticulospinal cultures containing hindbrain Chx10^+^ and HB9^+^ spinal motor neurons, we hypothesized that each cell type would develop a distinct pattern of network activity, which would be consistent with their distinct behavioral roles. Because motor neurons are controlled by reticulospinal neurons *in vivo*, we further hypothesized that the reticulospinal neurons’ activity would come to dominate in a combined coculture. This would support the idea that the electrical and biochemical properties of one neuronal subtype can drive the activity of the entire network *in vitro*.

Here, we report that Chx10^+^ hindbrain neurons develop synchronized network bursts that differ from the uncorrelated and irregular activity of HB9^+^ motor neurons, and that in coculture motor neurons are recruited into Chx10^+^ neuron bursts. We then further identify some synaptic mechanisms that drive these circuit dynamics.

## Materials and Methods

### Cell Culture

All cells were cultured at 37°C in 5% carbon dioxide and 95–100% humidity in Revco Ultima II CO_2_ incubators (Thermo Electron). Primary cortical glia were dissected and dissociated from Swiss Webster mice at P1-4 using the protocol described in Schildge et al. (2013). Mouse pups were anesthetized with 5% isoflurane for 5 min, then decapitated. The forebrain was separated from the cerebellum and midbrain. The corpus callosum was severed, then the meningeal covering was peeled away. Forebrain tissue was dissociated in 10% trypsin (0.25% EDTA, Gibco, 25200-056) and passed through a 35 μm filter (Corning, 352235). Cells were cultured on 100 mm cell culture dishes treated with 0.1% gelatin (ATCC, PCS-999-027) at a density of ∼5 × 10^4^ cells/cm^2^ and grown until confluent, usually within 8 days. Glial culture media contained high glucose DMEM (Sigma-Aldrich, 51441C), 10% heat inactivated fetal bovine serum (ATCC, SCRR-30-2020), and 1% penicillin/streptomycin/antimycotic (Sigma-Aldrich, A5955). Once the glia reached confluence, they were dissociated with trypsin and cultured on sterile 5mm n.1 glass coverslips (Warner, 640700) treated with 1 mg/ml Poly-D-Lysine (Millipore, A-003-E) and 1 mg/ml laminin (Corning, 354232) in 24-well plates at a density of 5 × 10^5^ cells/well. Neurons were seeded on this feeder layer of glia once it reached confluence, after about 8 days.

ES-cell derived motor neurons were generated using the protocol described in Wichterle et al. (2002) from the HBG3 ES cell line, in which enhanced green fluorescent protein (eGFP) is expressed under the control of the HB9 promoter (courtesy of Wichterle lab). ES cells were grown in ADFNK media that consisted of 1:1 DMEM/F12 (Millipore, DF-041-B): Neurobasal (Gibco, 21103049), 10% knock out serum replacement (Gibco, 10828010), 1% penicillin/streptomycin/antimycotic, and 1% GlutaMax supplement (Gibco, 35050061) for 2 days until they formed embryoid bodies. Media were supplemented on days 2 and 5 with 1 μM retinoic acid (Sigma-Aldrich, R2625) and 1 μM smoothened agonist (Calbiochem, 566661). On day 6, embryoid bodies were dissociated with papain according to manufacturer’s instructions (Worthington, LK003150).

Unsorted HB9^+^ motor neurons were plated on 5 mm glass coverslips in a 24-well plate on top of a feeder layer of glia at a density of 1 × 10^6^ cells/well. HB9^+^ neurons that underwent FACS sorting were plated on Poly-D-lysine and laminin coated 5 mm glass coverslips at a density of 5 × 10^5^ cells/well. For glial coculture, sorted HB9^+^ neurons were seeded on glass coverslips with a feeder layer of astroctyes at a density of 5 × 10^5^ cells/well. For multi-electrode recordings, standard 60-elecrode MEAs (MultiChannel Systems, 890276) were sterilized and then coated with poly-D-lysine and laminin and seeded with 1 × 10^6^ sorted HB9^+^ neurons. For glial coculture on MEAs, poly-D-lysine and laminin treated arrays were seeded with 5 × 10^5^ glial cells that were grown to confluence prior to seeding with 1 × 10^6^ sorted HB9^+^ neurons. Media consisted of the BrainPhys neuronal medium (StemCell, 5792) supplemented with 2% NeuroCult SM1 neuronal supplement (StemCell, 5711), 1% N2-supplement (Gibco, 17502048), 1% GlutaMax supplement, 1% pen/strep/antimycotic, 1 μM Adenosine 3′,5′-cyclic monophosphate, *N*^6^,*O*2′-dibutyryl-sodium salt (dbCaMP, Calbiochem, 28745), 10 ng/ml Brain derived neurotrophic factor (BDNF, MACS, 130-093-811), 10 ng/ml Glial derived neurotrophic factor (GDNF, GoldBio, 1170-14-10), and 1 μM ascorbic acid (Sigma-Aldrich, A4403). To produce HB9:GFP negative control for FACS sorting, ES-cell derived HB9^+^ motor neurons were generated in parallel from the E14 ES cell line (courtesy of Hatten lab).

Reticulospinal Chx10^+^ neurons were dissected from E12.5 mouse embryonic hindbrains using the protocol described in Fantin et al. (2013) from mice in which cyan fluorescent protein (CFP) is expressed under the control of the Chx10 promoter (Zhong et al., 2010). To produce the cells, a male mouse homozygous for Chx10:CFP (courtesy of Sharma lab) was mated with a Swiss Webster female mouse (Taconic). On day E12.5 of the pregnancy, the pregnant female was anesthetized in 5% isofluorane and oxygen and euthanized via cervical dislocation.

For the hindbrain dissection, each embryo was decapitated just rostral to the forelimb and the neural tube was isolated from the rest of the tissue. The developing rhombencephalon (hindbrain) segment corresponding to the position of the reticular formation in adults was excised and trimmed at the rostral and caudal ends. Dissections were performed in ice cold HBSS buffer (Gibco, 14175-095) supplemented with 1% pen/step/antimycotic, 20 mM D-glucose (Sigma-Aldrich, G8769), and 1 μM ascorbic acid. Hindbrains were dissociated with papain and sorted using flow cytometry to isolate the Chx10^+^ subpopulation. To produce Chx10:CFP negative control for FACS sorting, E12.5 hindbrains were derived from Swiss Webster mouse embryos. Sorted Chx10^+^ hindbrain neurons were seeded on either 5 mm glass coverslips in a 24-well plate or MEAs, both prepared with a confluent layer of glia, at a density of 1 × 10^4^ neurons/well of coverslips or 4 × 10^4^ neurons/array. All Chx10^+^ hindbrain neurons were cultured in Neurobasal medium supplemented with 2% SB-27 (Gibco, 17504044), 1% GlutaMax, 1% pen/strep/antimycotic, 1 μM dbCaMP, 10 ng/ml BDNF, 10 ng/ml GDNF, and 1 μM ascorbic acid.

For reticulospinal cocultures, sorted HB9:GFP^+^ motor neurons and Chx10:CFP^+^ hindbrain neurons were seeded together on a confluent layer of glia on either 5 mm coverslips or MEAs. On coverslips in a 24-well plate, HB9^+^ neurons were seeded at a density of 2.5 × 10^5^ cells/well and Chx10^+^ neurons were seeded at a density of 1 × 10^5^ cells/well. On MEAs, HB9^+^ neurons were seeded at a density of 1 × 10^6^ cells/array and Chx10^+^ neurons were seeded at a density of 4 × 10^5^ cells/array. Cocultures were grown in the same supplemented BrainPhys medium used for HB9^+^ cultures.

### Animals

Mice were group housed in a 12-h light/dark schedule, with food and water provided *ad libitum*. For timed matings, two females were introduced into the home cage of a single male, where they remained for the duration of the mating. Females were checked for vaginal plugs every 24 h and removed to separate cages after plug was detected, and singly housed for the duration of the timed pregnancy. All animal procedures and protocols were approved by the Rockefeller Institutional Animal Care and Use Committee.

### Flow Cytometry

All samples were sorted on the basis of fluorescent marker expression on the BD FACSAriaII benchtop flow cytometer with a 100 μm nozzle and 20 psi sheath pressure. Flow cytometry was performed at the Flow Cytometry Resource Center at Rockefeller University.

To isolate HB9:GFP^+^ motor neurons, embryoid bodies derived from HBG3 ES cells were dissociated on day 6 using papain and resuspended in FACS buffer for embryoid bodies that contains phenol-free HBSS supplemented with 2% heat-inhibited horse serum (Gibco, 26050088) and 5 U/mL DNAse (Worthington, LK003172). For the GFP negative control, embryoid bodies were derived from E14 ES cells and prepared under parallel conditions. Between 10 and 20 nM DAPI (Invitrogen, D1306) was added to each sample as a dead-cell exclusion dye. Each sample was excited by a violet 405 nm laser and dead cells were excluded on the basis of emission in the DAPI wavelength 461 nm using the 405D filter. Single cells were distinguished from doublets on the basis of forward and side scatter of the sample comparing the scatter area versus width. GFP fluorescence was detected using illumination from a 488 nm blue laser equipped with a 535/30 nm filter and the gate for GFP^+^ cell isolation was set based on a comparison of the GFP fluorescence of the HBG3-derived sample and the E14-derived sample. Typically, 50–60% of input cells from HBG3-derived embryoid bodies expressed GFP.

For Chx10:CFP^+^ hindbrain neurons, hindbrains from heterozygous Chx10::CFP^+/−^ mice were dissociated at E12.5 using papain and resuspended in FACS buffer for hindbrains that contained high glucose phenol-free DMEM supplemented with 10% heat-inactivated fetal bovine serum, 1% pen/strep/antimycotic, and 5 U/ml DNAse. For the CFP-negative control, hindbrains from Swiss Webster mice were prepared under parallel conditions. Approximately 20 nM ToPro3 (Invitrogen, T3605) was added to each sample as a dead cell exclusion dye. Each sample was excited by a red 640 nm laser, dead cells were excluded on the basis of emission in the ToPro3 wavelength using the 640C 670/30 nm filter. As with the HB9:GFP^+^ motor neurons, single cells were distinguished from doublets on the basis of forward and side scatter area versus width. CFP fluorescence was detected using illumination from a 445 nm blue violet laser equipped with a 490/30 nm filter and the gate for CFP^+^ cell isolation was set based on a comparison of the CFP fluorescence of the Chx10:CFP^+/–^-derived sample and the Swiss Webster-derived sample. Typically, 2.5–3% of input cells from Chx10:CFP^+/–^ mouse hindbrains expressed CFP.

### Electrophysiology

Coverslips containing neurons cultured on a feeder layer of astrocytes as described above (see Cell Culture methods) for 5–10 days were perfused with 1x HEPES-ACSF in the recording chamber (HEPES-ACSF: 135 mM NaCl, 10 mM HEPES, 10 mM glucose, 5 mM KCl, 1 mM CaCl_2_-2H_2_O, 1 mM MgCl_2_) under constant flow (∼5 ml/min). All cells were patched using pulled glass pipettes with an R_*E*_ of 5 to 12 MΩ filled with a standard internal pipette solution (K-gluconate: 14 mM, HEPES-K: 10 mM, NaHCO_3_: 60 μM, Mg-ATP: 4 mM, Na_2_-ATP: 2 mM, Na-GTP: 30 μM, sucrose: 8 mM, CaCl_2_: 1 mM, EGTA: 5 μM). Data were acquired on the MultiClamp 700B (Axon instruments) using ClampEx software. HB9^+^ spinal motor neurons were identified by GFP signal imaged using an Olympus BXS1W1 upright fluorescence microscope equipped with a FITC/EGFP filter (480/535nm ex/em, Chroma). Chx10^+^ hindbrain neurons were identified by CFP signal from an ECFP filter (436/480 nm ex/em, Chroma).

Once a giga-seal was achieved, the membrane voltage of the neuron was recorded for 1 min at 1 kHz sampling frequency without injecting additional current to measure the spontaneous activity of the neuron. For current-clamp experiments, current was injected to bring V_m_ to −70 mV and current steps were applied in 10 pA increments from −10 to 130 pA for 1 s duration, returning to −70 mV holding potential between steps. For voltage clamp experiments, the cell was held at −80 pA for 100 ms before stepping voltage injection from −100 to 150 mV in 10 mV increments for 100 ms, returning to the −80 pA holding potential between each step.

Data analysis and plotting of patch clamp data were performed using ClampFit and Matlab (see github.com/abubnys/patch_clamp_analysis for specific scripts used). To generate IV plots of voltage-gated sodium current from voltage-clamp data, the local minimum evoked current within 30 ms of voltage step onset was subtracted from the mean current during the last 30 ms of the voltage step and plotted against the magnitude of the injected voltage.

### Multi-Electrode Recordings

Multi-electrode arrays were cultured with HB9^+^ motor neurons or Chx10^+^ hindbrain neurons as described above (see Cell Culture methods). For the duration of the lifetime of the culture (D3 to D30 days after plating for Chx10^+^ and D7 to D30 days after plating for HB9^+^ neurons), spontaneous extracellular activity was recorded using the MEA2100-Lite system (MultiChannel Systems). The array was placed in the recording apparatus and allowed to equilibrate at room temperature for 30 min prior to recording for 4 min. Data acquisition was performed on MCRack with an input voltage range of −19.5 to +19.5 mV and a sampling frequency of 20 kHz. Raw electrode data for 60 electrodes were processed through a Bessel 4th order high pass filter with a cutoff at 400 Hz. The spike detection threshold was 5 standard deviations below the mean of the filtered recordings. Raw and filtered data, along with spike timestamps were converted to.txt files using MC_DataTool and the resulting files were analyzed in Matlab.

For wash-in experiments of the synaptic blockers 6-cyano-7-nitroquinoxaline-2,3-dione disodium salt (CNQX), D-(-)-2-amino-5-phosphonopentanoic acid (AP5), and bicuculline on the MEAs, warmed 1x HEPES-ACSF was perfused through the MEA after the 30-min equilibration period at ∼5 ml/min for 10 min. The baseline activity of the MEA was recorded for 2 min under the previously mentioned parameters. Then, 100 μL of a 10x solution of the drug was slowly perfused in at 50 μL/min for 2 min while recording. After 2 min the pump was stopped and the steady-state activity of the array in the presence of the drug was recorded for 4 min. The MEA was washed with 1x HEPES-ACSF at 5 ml/min for 10 min in between drug applications. Filtered electrode data were converted using MC_DataTool and analyzed in Matlab. The final concentrations of the drugs used were 20 μM CNQX (Tocris, 479347-85-8), 50 μM AP5 (Tocris, 79055-68-8), and 60 μM bicuculline (Tocris, UN1544).

Data analysis for perfusion experiments was performed in Matlab (see github.com/abubnys/MEA_perfusion_package for specific scripts used). For initial spike data extraction, the high-pass filtered recordings from each electrode generated by MC_Rack (multichannel systems, Reutlingen, Germany) were converted into .txt format using MC_DataTool (multichannel systems). Spikes were detected in each channel using a manual threshold adjusted to pick up deviations that were approximately five standard deviations below the baseline of the recording and analysis of spike waveforms was used to determine whether one or more neurons was contributing to the observed signal. Spike sorting was performed on these data by plotting the aggregate collection of waveforms from recorded spikes. If this collection of waveforms fell within multiple visually distinguishable distributions, manual thresholds for each distribution were set by drawing a line through the waveforms visually classified as similar and then categorizing all recorded spikes according to whether they cross this threshold line or not. Then, spike rate was calculated for each waveform type by counting the number of spikes that fall within bins of 100ms width and multiplying by 10 to covert to units of Hz.

To facilitate comparison of different spike rates across all recordings, spike rates were smoothed using a spline function, binned according to the average spike rate in non-overlapping 10 s intervals, then normalized to set the average spike rate from the first 10 bins (corresponding to the first 100 s of recording) to 1.

To determine whether synaptic blocker wash-in had a dose-dependent effect on the activity of each culture type, normalized spike rate data from each electrode on the MEA that recorded spontaneous neuronal activity were pooled across all drug wash-in trials for a given culture type. The data corresponding to the period when the drug was washed in (2–4 min into the recording) were fit to a linear mixed effects model using the function fitlme() in Matlab with the normalized spike rate as the predictor variable and electrode as the random effect:

Drug⁢dose⁢(μ⁢M)∼1+normalized⁢spike⁢rate+(1|electrode)

### Calcium Imaging

HB9^+^, Chx10^+^, or combined cocultures grown on 5 mm coverslips with a feeder layer of glia were loaded with Rhod-3 AM dye according to the manufacturer’s instructions (Molecular Probes, R10145), then washed with 1x HEPES-ACSF. Calcium imaging was subsequently performed in 1x HEPES-ACSF.

For Chx10^+^ cultures, calcium reporter dye fluorescence during spontaneous activity was imaged using an inverted spinning disk confocal microscope (Zeiss Axiovert 200) equipped with an EMCCD camera (Andor iXon). Solid state lasers were used for excitation at 443 and 561 nm (Spectral Applied) paired with a polychroic filter with 440, 491, 561, and 640 nm filters. Imaging acquisition was performed using MetaMorph software. Chx10^+^ neurons were identified by CFP signal (440/480 nm ex/em) and rhodamine3 signal was identified on the Texas Red channel (561/620-60 nm ex/em). Calcium imaging data were acquired via time-lapse, with a 150 ms interval and 100 ms exposure time for 2 min.

For HB9^+^ cultures, HB9/Chx10 cocultures, and astrocyte cultures, spontaneous calcium activity was imaged at room temperature and ambient CO_2_ using an Olympus BXS1W1 upright fluorescence microscope equipped with an Evolution QEi digital CCD camera (MediaCybernetics). A 120W mercury vapor short arc bulb was used as the fluorescence light source (X-Cite series 120Q). Imaging acquisition was done using NIS-Elements BR software. Hb9^+^ spinal motor neurons were identified by GFP signal using a FITC/EGFP filter (480/535 nm ex/em, Chroma) and imaged with an exposure time of 100 ms.

Chx10^+^ hindbrain neurons were identified by CFP signal using an ECFP filter (436/480 nm ex/em, Chroma) and imaged with an exposure time of 100ms. Rhodamine3 signal was imaged using a CY3/TRITC filter (545/605 nm ex/em, Chroma) with an exposure time of 60 ms per frame for 40–80 s.

For experiments involving application of the AMPA_R_ blocker CNQX, the spontaneous calcium activity of HB9/Chx10 cocultures in HEPES-ACSF solution was imaged to determine a baseline level of activity. Then, 200 μl of a 100x solution of CNQX was injected into the bath for a final drug concentration of 40 μM. The culture was allowed to equilibrate for 5 min before imaging of spontaneous calcium activity in the presence of the drug. The drug was washed out by replacing 50% of media with fresh HEPES-ACSF in 5 repeated washes, then the culture was allowed to equilibrate for 5 min before measuring recovery of spontaneous activity.

Calcium imaging data for all experiments were analyzed in Matlab (see github.com/abubnys/calcium_imaging_ ROI_analysis for specific scripts used). Due to overlap between CFP and GFP emissions spectra, CFP^+^ neurons appear on the GFP fluorescence channel and were distinguished from HB9:GFP^+^ neurons on the basis of their fluorescence on the CFP channel. ROIs were manually drawn around the cell bodies of identified CFP^+^ and GFP^+^ neurons and the mean Rhodamine3 fluorescence within the ROI was calculated at each frame of the recording in the Rhod3 channel.

Editing of rhodamine3 fluorescence time-course videos was performed in Fiji. For each video, brightness and contrast was adjusted uniformly across the image stack using the “auto” adjust function. Then, the minimum intensity for each pixel across the image stack (calculated using the “Z-project, minimum” function) was subtracted from each image in the stack to remove noise. Then, brightness and contrast were adjusted again across the image stack. Video playback is 20fps.

### Statistical Methods

All statistical analyses were performed in Matlab. Results are presented as mean ± SEM. The statistical significance level for all of these analyses was set to *p* < 0.05. For patch clamp experiments, age matched HB9:GFP^+^ neurons from cultures that were either immediately plated after dissociation from embryoid bodies or underwent flow cytometry prior to plating were subjected to the same battery of current clamp, voltage clamp, and spontaneous activity recordings. The specific codes used to analyze and plot data from the patch clamp experiments can be found at github.com/abubnys/patch_clamp_analysis. Student’s *t*-test was used for between-group comparisons of voltage-gated I_Na_ and spike threshold.

For MEA recordings, raw data was passed through a Bessel 4th order high pass filter with a cutoff of 400 Hz to filter out noise, and a threshold of 5 standard deviations below the mean was used to detect spikes from the filtered recordings. Cross-correlation, using the Matlab function xcorr(), was used to determine the degree of coordination of spikes across electrodes that detected spontaneous activity from each recording. For synaptic blocker experiments, all electrodes with spontaneous activity from MEA recordings were pooled according to culture type and normalized. To determine if there was a dose-dependent effect of synaptic blocker on spike rate, this data was fit to a linear mixed effects model. The specific codes used to process and analyze data from the perfusion experiments can be found at github.com/abubnys/MEA_perfusion_package.

For calcium imaging experiments, ROIs were manually drawn around neurons identified on the basis of CFP and GFP fluorescence to be Chx10^+^ or HB9^+^ and the mean rhodamine calcium indicator fluorescence was calculated within each ROI over the time course of the recording. The specific codes used to process and analyze calcium imaging data can be found at github.com/abubnys/calcium_imaging_ROI_analysis. To facilitate plotting of calcium imaging data in Figures 3–5, these calcium activity traces were further normalized to a level baseline that takes into account gradual photobleaching over the course of the experiment. The baseline of each calcium trace, calculated by passing the raw data through a smoothing spline function using the Matlab fit() function with a smoothing parameter of 0.0001, was subtracted from the raw data. Then, each trace was normalized along the 0-to-1 scale to take into account arbitrary differences between the overall fluorescence of different cells.

### Code Accessibility

All custom written code used for this study is available on github. The code used to analyze and visualize patch clamp data is available at github.com/abubnys/patch_clamp_analysis. Code for quantifying calcium imaging data is available at github.com/abubnys/calcium_imaging_ROI_analysis. The code used for extracting and analyzing data from synaptic blocker perfusion experiments on MEAs is available at github.com/abubnys/MEA_perfusion_package.

## Results

### Developing Reticulospinal Cultures

Numerous studies of mixed populations of neurons from various brain regions including cortex, amygdala, and spinal cord have demonstrated a strong tendency to develop network bursts when cultured on MEAs. These bursts occur when many neurons across the cultured network fire at once at regular intervals (Van Pelt et al., 2005; Wagenaar et al., 2006; Dauth et al., 2016; Black et al., 2017). The generation of such bursts is the product of a precise balance between different ionic conductances within individual neurons (Golowasch et al., 1999; Marder et al., 2014) and interactions between classes of excitatory and inhibitory interneurons that work in concert to balance network activity between a state of excitation and complete quiescence (Li et al., 2009; Maheswaranathan et al., 2012; Sternfeld et al., 2017).

We sought to test whether networks of excitatory neurons could generate coordinated bursts in the absence of inhibitory interneurons by purifying neuronal subpopulations, which allowed us to culture identified neurons at defined stages of development. We focused specifically on reticulospinal cultures containing homogeneous populations of HB9^+^ spinal motor neurons and hindbrain Chx10^+^ neurons. Hindbrain Chx10^+^ neurons are known to play a role in regulating locomotor gait and breathing rhythm and they have descending projections to the spinal cord (Crone et al., 2012; Bretzner and Brownstone, 2013; Bouvier et al., 2015). Spinal motor neurons provide direct limb muscle innervation. Thus, the *in vivo* function of both neuronal subtypes predisposes them to rhythmic bursts.

**FIGURE 1:**
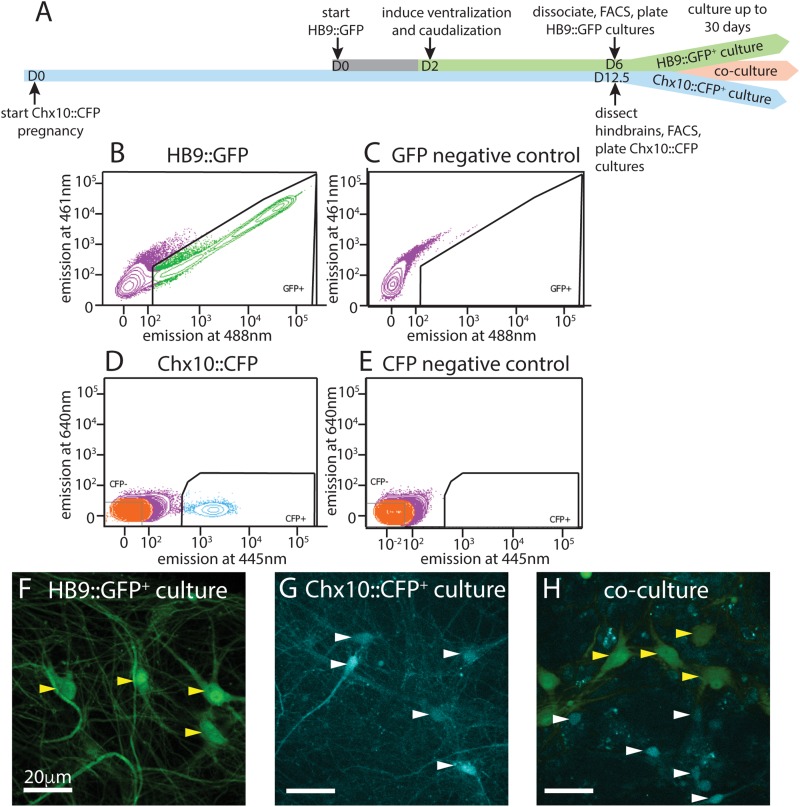
Isolation and culture of HB9^+^ motor neurons and Chx10^+^ hindbrain neurons. **(A)** Timeline schematic of isolating and setting up HB9:GFP^+^, Chx10:CFP^+^, and combined co-cultures. **(B–E)** Sample FACS plots and thresholds for isolation of HB9:GFP^+^ and Chx10:CFP^+^ neurons. **(B)** GFP^+^ neurons from HB9:GFP stem-cell derived embryoid bodies after 6 days in culture (DIC) **(C)** embryoid bodies derived from non-transgenic ES cells (negative control) **(D)** CFP^+^ neurons from E12.5 hindbrains of Chx10:CFP mice and **(E)** Swiss Webster mice (negative control). **(F–H)** Fluorescent photomicrographs of neurons cultured after sorting. Yellow arrowheads indicate HB9:GFP^+^ neurons and white arrowheads indicate Chx10:CFP^+^ neurons. **(F)** Sorted HB9:GFP^+^ neurons, 16 DIC. **(G)** sorted Chx10:CFP^+^ hindbrain neurons, 10 DIC (scale bar 20 μm) **(H)** combined culture of both subtypes, 16 DIC (scale bar 20 μm).

To isolate pure populations of HB9^+^ spinal motor neurons and hindbrain Chx10^+^ neurons, we employed fluorescence activated cell sorting (FACS). We cultured these cell types as single populations and also as a mixed reticulospinal culture (Figure 1A). We differentiated HB9^+^ spinal motor neurons from HBG3 embryonic stem cells using Wichterle et al.’s (2002) protocol to induce the spinal motor neuron identity (Wichterle et al., 2002). We note that a second population of HB9^+^ interneurons with rhythm generating function distinct from motor neurons exists in the intact spinal cord, but previous studies have demonstrated that these ChAT^–^ interneurons constitute < 5% of HB9^+^ neurons generated by the program of ventralization and caudalization used here (Wichterle et al., 2002; Miles et al., 2004; Di Giorgio et al., 2007; López-González et al., 2009).

Following the motor neuron differentiation, embryoid bodies were dissociated 6 days after formation and sorted on the basis of HB9:GFP expression. E14 stem cells lacking GFP were used as a negative control for FACS (Figures 1B,C). Approximately 50–60% of unsorted cells in the embryoid body derived from HBG3 ES cells expressed GFP. FACS sorting for GFP expression enriched this population to >96% purity. HB9:GFP^+^ motor neurons were subsequently cultured on a layer of cortical astrocytes to improve axonal outgrowth and network development (Figure 1F).

**FIGURE 2:**
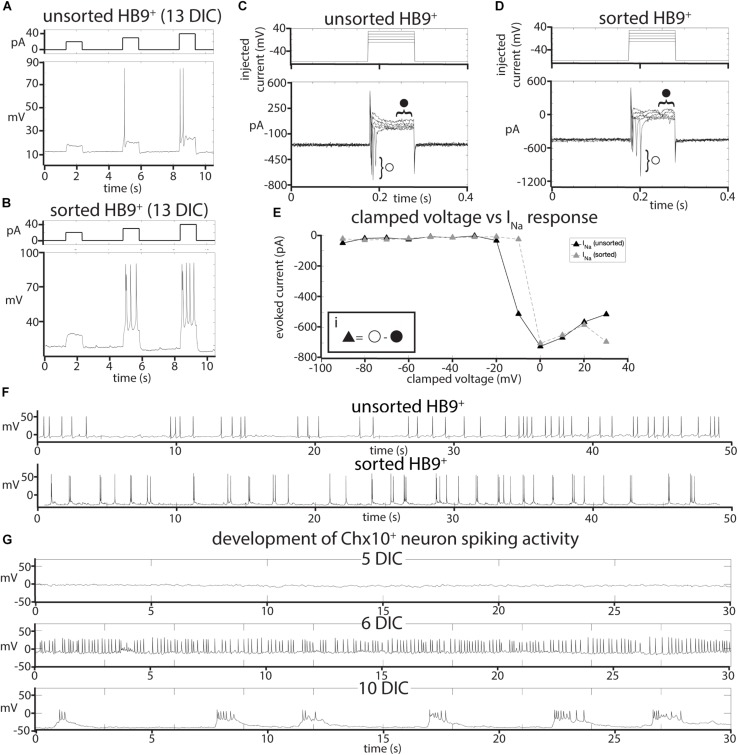
Effects of FACS sorting on neuron electrophysiology. **(A,B)** Comparison of the response of **(A)**, unsorted and **(B)**, sorted HB9:GFP^+^ neurons (bottom panels) to injections of 20, 30, and 40 pA current (top panels). **(C,D)** Response of **(C)**, unsorted and **(D)**, sorted HB9:GFP^+^ neurons (bottom panels) to voltage step injection of –90 to 30 mV (top panels, result for –10 to 30 mV injections shown) (7 DIC). Sodium current (I_Na_) was calculated at each injected voltage step by subtracting the steady state current response (solid circle) from the initial current minimum (empty circle) (formula shown in insert *Ei*). **(E)** I-V plot of voltage-gated Na^+^ currents for sorted and unsorted HB9:GFP^+^ neurons calculated from the voltage clamp experiment results shown in **(C,D)**. **(F)** Spontaneous activity of unsorted (top panel) and sorted (bottom panel) HB9:GFP^+^ cells at 13 DIC. **(G)** Spontaneous activity of sorted Chx10:CFP^+^ neurons at 5 DIC (top), 6 DIC (middle) and 10 DIC (bottom).

To test whether sorting affected the electrophysiological activity of HB9^+^ neurons, we performed whole cell patch clamp on HB9:GFP^+^ neurons from sorted and unsorted cultures grown in parallel under identical conditions. After 7 days in culture, HB9^+^ neurons in both treatments responded to brief current pulses with spike trains, having a spike threshold around 20 pA (Figures 2A,B). They developed voltage gated sodium current (I_Na_) with maximum current evoked at −2 ± 12 mV (Figures 2C–E) that was not significantly different between sorted and unsorted populations (Student’s two-tailed *T*-test *p* = 0.879). After 13 days in culture, both sorted and unsorted HB9^+^ motor neurons also developed spontaneous spike trains (Figure 2F).

We then isolated and cultured primary hindbrain neurons expressing the transcription factor Chx10, also using the FACS approach. We first assessed the Chx10^+^ neurons’ behavior *in vitro* as a homogeneous population, and then in combination with HB9^+^ neurons to determine if they could form a reticulospinal circuit *in vitro*. For these experiments, we dissected neurons from embryonic Chx10:CFP^+/–^ mice at E12.5, prepared a single cell suspension and used FACS to isolate the CFP^+^ population. As a negative control for CFP expression, we used hindbrains taken from wildtype (WT) Swiss Webster E12.5 mouse embryos that do not express CFP (Figures 1D,E).

The hindbrains contained 2–3% Chx10:CFP^+^ neurons, and sorting enriched this population to >95% purity. These CFP^+^ neurons were then cultured on a layer of cortical astrocytes, which is known to improve the development and long-term viability of neuronal cultures (Figure 1G) (Wang et al., 1994; Maher et al., 1999; Boehler et al., 2007).

It is possible that, when removed from the intact reticular formation with its descending inputs and diversity of other cell types, Chx10^+^ hindbrain neurons would not develop any intrinsic activity that could pattern a reticulospinal circuit. To assess the electrophysiological development of sorted Chx10^+^ neurons, we used whole-cell patch clamp to record the spontaneous activity of single cells in cultures at different ages ranging from 1 to 30 days in culture. For Chx10^+^ hindbrain neurons, the measured membrane capacitance was 22.75 ± 2pF, membrane resistance was 787.27 ± 105 MΩ, access resistance was 29.01 ± 3 MΩ, and membrane voltage was −22.6 ± 4 mV. We found that Chx10^+^ hindbrain neurons developed spontaneous electrophysiological activity after 5 days in culture. This activity started off as random trains of spikes, but gradually became organized into robust, regular bursts by 10 days in culture and this pattern of activity continued throughout the remaining lifetime of the cultures (Figure 2G).

### Motor and Chx10 Neuron Cultures Develop Distinct Patterns of Network Activity

Having established that HB9^+^ motor neurons and Chx10^+^ hindbrain neurons develop spontaneous electrophysiological activity at the single cell level, we sought to determine whether cultures of either cell type, which are composed almost exclusively of excitatory neurons and astrocytes, could generate spontaneous patterns of network activity, whether these patterns would organize into network bursts, and whether there were any cell-type specific differences in such activity.

To record the activity of multiple neurons at different time points, we cultured sorted HB9^+^ motor neurons on multielectrode arrays (MEAs) containing a grid of 64 extracellular recording electrodes (Figure 1H). We recorded their spontaneous activity daily over 30 days, starting from the day after plating.

**FIGURE 3:**
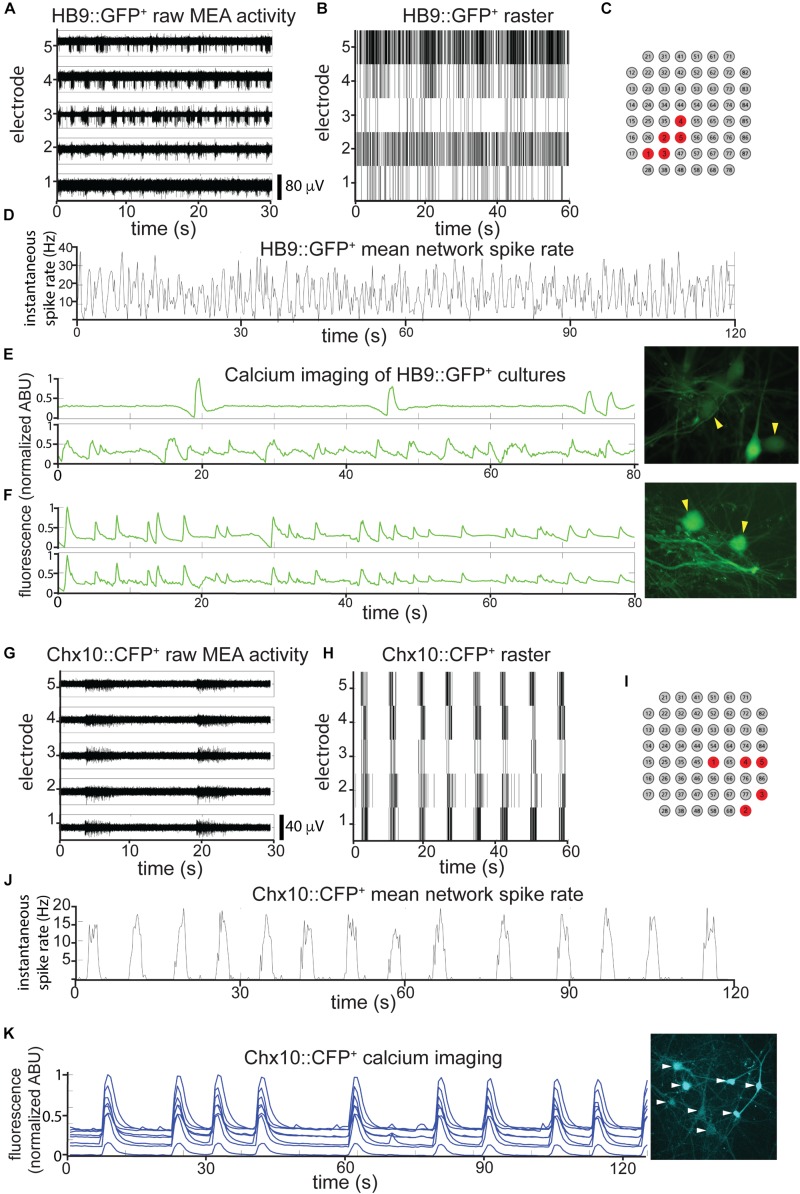
HB9^+^ motor neurons and Chx10^+^ hindbrain neurons develop different patterns of activity *in vitro*. **(A–C)** Example of a multielectrode array (MEA) recording of sorted HB9:GFP^+^ neurons (18 DIC), both as **(A)**, high pass filtered MEA data (*y*-axis scale bar on bottom right) and **(B)**, raster plot. Locations of the electrodes shown are indicated in red in **(C)**. **(D)** Mean spike rate of entire HB9:GFP^+^ neuron culture from **(A,B)**. **(E,F)** Quantification of calcium-sensitive Rhodamine3 dye fluorescence in the cell bodies of HB9:GFP^+^ neurons **(E)**, 19 DIC, see Supplementary Video S2 for rhodamine fluorescence time course, **(F)**, 32 DIC, see Supplementary Video S3. Corresponding right panels are photomicrographs of neurons quantified for calcium activity, indicated by yellow arrowheads. **(G–I)** Example of a multielectrode array (MEA) recording of sorted Chx10:CFP^+^ neurons (5 DIC) as **(G)**, high-pass filtered MEA data (*y*-axis scale bar on bottom right) and **(H)**, raster plot. locations of electrodes on array in red in **(I)**. **(J)** Mean spike rate of entire Chx10:CFP^+^ neuron culture from **(G,H)**. **(K)** Calcium imaging of Chx10:CFP^+^ neurons (10 DIC), also shown in Supplementary Video S4. Right panel is a photomicrograph of identified neurons quantified for calcium activity, indicated by white arrowheads.

We found that on their own, without astrocytes, sorted HB9^+^ motor neurons did not develop any spontaneous activity on the MEA (*n* = 6). However, when these neurons were cultured on a confluent layer of astrocytes, they gradually developed robust network activity that remained stable over a month of recording (*n* = 14). We note that astrocytes cultured on their own did not develop spontaneous activity when recorded on MEAs (*n* = 3), although we did observe spontaneous calcium flux in astrocyte cultures visualized with the calcium-sensitive dye Rhodamine3 (Supplementary Video S1). The activity of HB9^+^ motor neuron/astrocyte cultures was not well coordinated, even among neighboring recording electrodes (Figures 3A–C). To assess whether the overall activity of the culture had a hidden underlying temporal structure, we calculated the mean spike rate across all active channels of the HB9^+^ motor neuron cultures and found that it remained constant throughout the recording session (Figure 3D).

The patterns that we observed for neurons cultured on MEAs were consistent with widespread activation that recruits many neurons across the network. To determine what fraction of the neurons in each culture were contributing to overall activity, we used calcium imaging with the calcium-sensitive dye Rhodamine3 to assess HB9^+^ motor neuron activity with single-cell resolution. We observed randomly distributed calcium spikes that were asynchronous between neighboring neurons (Figure 3E and Supplementary Video S2), though more mature cultures did develop some synchrony (Figure 3F and Supplementary Video S3). The mean correlation coefficient between the spike rates of multiple neurons within the same HB9^+^ neuron culture was 0.15 ± 0.17 (*p* = 0.15).

When we cultured Chx10^+^ hindbrain neurons on MEAs with a confluent layer of astrocytes, we observed the emergence of spontaneous activity with these neurons as well. Unlike HB9^+^ neurons, Chx10^+^ neurons developed robust and coordinated network bursts (Figures 3G–J). Practically no spikes occurred outside of these sharply delineated bursting periods. The time between bursts (inter-burst interval) varied between 2 and 10 s throughout the lifetime of the cultures, with no apparent long-term trend. We observed the same sort of robust network bursts in Chx10^+^ hindbrain neuron cultures that recruited all cells visualized with calcium imaging (Figure 3K and Supplementary Video S4).

### HB9 and Chx10 Neurons Develop Correlated Activity in Coculture

Despite their common excitatory identity, we observed that HB9^+^ and Chx10^+^ hindbrain neurons develop distinct patterns of spontaneous network activity. If these two cell types fail to form functional connections to one another *in vitro*, these patterns of activity should remain unchanged in coculture, but if a unidirectional functional connection forms between Chx10^+^ and HB9^+^ neurons, we might expect to see one activity pattern dominate in coculture. To test these possibilities, we cultured the two cell types together as a mixed population on MEAs and recorded their spontaneous activity daily over 30 days. Since the survival of Chx10^+^ and HB9^+^ neurons after FACS sorting was highly dependent on their initial plating concentration, we cocultured these neurons at concentrations that were empirically determined to optimize the survival of each cell type, a ratio of 5:2 HB9^+^ to Chx10^+^ neurons.

**FIGURE 4:**
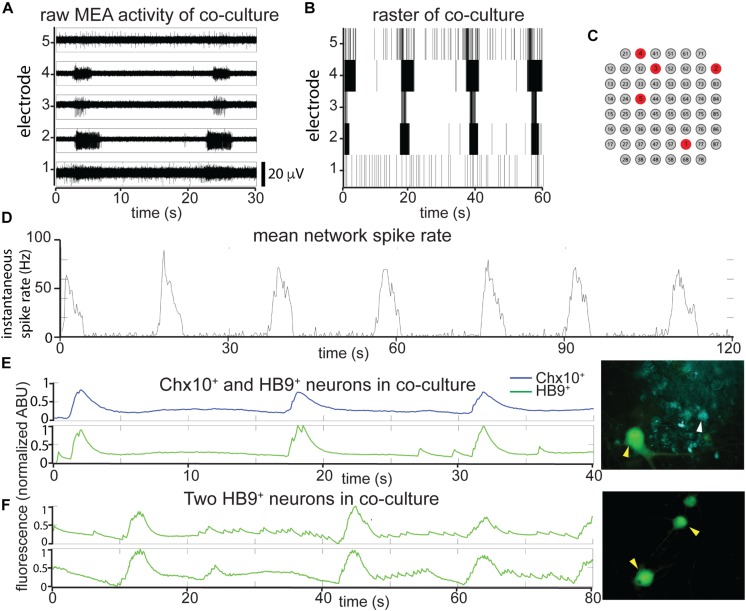
In reticulospinal coculture, Chx10^+^ hindbrain neurons drive patterned HB9^+^ neuron activity. **(A,B)** Example of an MEA recording of HB9:GFP^+^/Chx10:CFP^+^ neuron coculture (8 DIC), both as **(A)**, high pass filtered MEA data (*y*-axis scale bar on bottom right) and **(B)**, raster plot. Locations of the electrodes shown are indicated in red in **(C)**. **(D)** Mean spike rate of entire coculture from **(A,B)** over the course of 120 s. **(E,F)** Calcium imaging of neurons in co-culture. **(E)** Normalized calcium-sensitive fluorescence intensity over time in cocultured HB9:GFP^+^ and Chx10:CFP^+^ neurons participating in coordinated bursts, shown also in Supplementary Video S5. **(F)** Normalized calcium-sensitive fluorescence intensity of two HB9:GFP^+^ neurons from coculture (Chx10:CFP^+^ neurons not pictured) participating in network bursts, shown also in Supplementary Video S6. Corresponding right panels indicate identified neurons quantified for calcium activity, white arrowheads for Chx10:CFP^+^ and yellow arrowheads for HB9:GFP^+^.

Such cocultures develop spontaneous bursts of comparable time scale and duration to pure Chx10^+^ cultures, though some neurons continue to have spiking activity that resembles HB9^+^ motor neurons in between network bursts (Figures 4A–C). When the overall network activity was measured by averaging spike rates across all active electrodes, the Chx10-like network bursts predominated (Figure 4D).

It is possible that the bursts we observed in the reticulospinal culture were generated only by the Chx10^+^ neurons in the dish and that the HB9^+^ motor neurons were quiescent and did not contribute to network activity. In order to determine which cell type participates in the cultures’ network bursts, we used calcium imaging to obtain single cell resolution recordings of the coculture. We found that neighboring HB9^+^ and Chx10^+^ neurons both participate in network burst events (Figure 4E and Supplementary Video S5). Some HB9^+^ motor neurons in coculture also have brief, non-coordinated calcium spiking events that occur between the larger bursts (Figure 4F and Supplementary Video S6).

The percentages of Chx10^+^ and HB9^+^ neurons from calcium-imaging experiments that were spiking, bursting, both spiking and bursting, or inactive in each of the culture conditions are summarized in Table 1.

**TABLE 1:** Overview of activity patterns of Chx10^+^ and HB9^+^ neurons from calcium imaging experiments.

**Cell type**	**Number total cells recorded**	**Spiking: number (percentage)**	**Bursting: number (percentage)**	**Spiking and Bursting: number (percentage)**	**No activity: number (percentage)**
Chx10^+^ (co-culture)	125	16 (13%)	64 (51%)	17 (14%)	28 (22%)
Chx10^+^ (alone)	483	0	312 (65%)	7 (1%)	164 (34%)
HB9^+^ (co-culture)	67	13 (19.5%)	13 (19.5%)	29 (43%)	12 (18%)
HB9^+^ (alone)	176	81 (46%)	0	2 (1%)	93 (53%)

### HB9^+^ and Chx10 Network Activity Is an AMPA Receptor-Dependent Process

**FIGURE 5:**
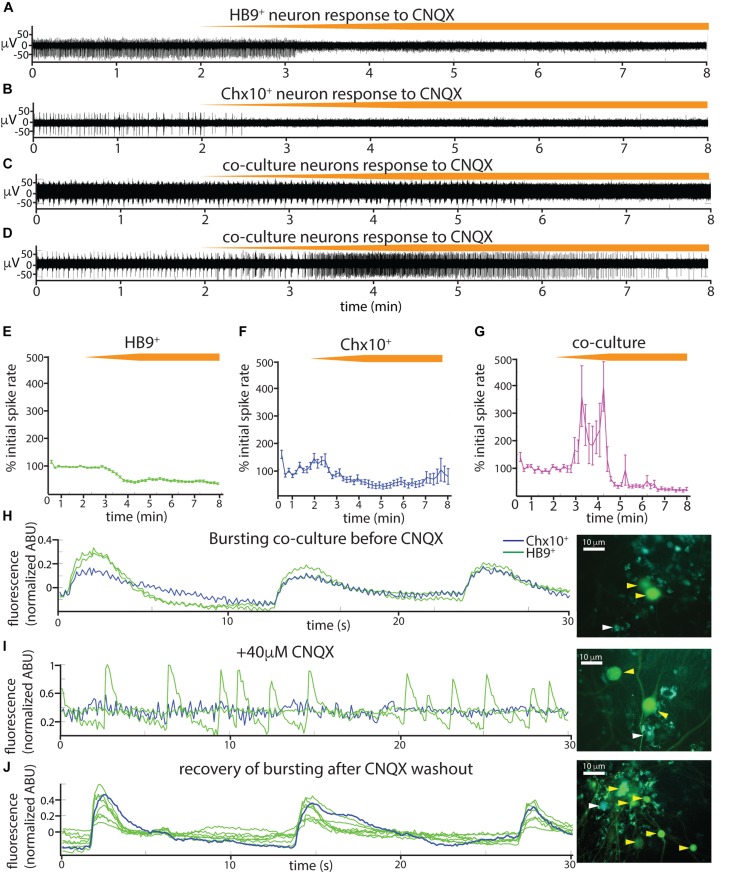
Spontaneous activity in reticulospinal cultures is an AMPA_R_-dependent process. **(A–D)** Examples of high pass filtered MEA recordings of spiking neurons during wash-in of a 200 μM solution of the AMPA_R_ blocker CNQX at 50 μL/min (final CNQX concentration 20 μM), orange bars show time course of blocker wash-in. **(A)** Neuron from HB9:GFP^+^ culture, **(B)** neuron from Chx10:CFP^+^ culture, **(C,D)** examples of two different kinds of responses to CNQX of neurons from HB9:GFP^+^/Chx10:CFP^+^ coculture. **(E–G)** Normalized mean responses of all neurons recorded from electrodes with activity to CNQX wash-in, **(E)** HB9:GFP^+^ cultures (*n* = 3), **(F)** Chx10:CFP^+^ cultures (*n* = 3), **(G)** HB9:GFP^+^/Chx10:CFP^+^ cocultures (*n* = 4). **(H–J)** Calcium imaging of coculture **(H)**, bursting prior to CNQX application (shown also in Supplementary Video S7), **(I)** inhibition of bursting, but not HB9:GFP^+^ spiking, by application of 40 μM CNQX (shown also in Supplementary Video S8), and **(J)** bursting recovers after washout of CNQX (shown also in Supplementary Video S9). Corresponding right panels are photomicrographs of neurons quantified for calcium activity, indicated by white arrowheads for Chx10:CFP^+^ and yellow arrowheads for HB9:GFP^+^.

The spontaneous coordinated activity we observed in Chx10^+^ and HB9^+^ neuron cultures could be the product of intrinsic pacemaker properties of these neurons or an emergent property of the network that is dependent on synaptic transmission. To distinguish between these alternatives, we applied a panel of synaptic blockers targeting α-amino-3-hydroxy-5-methyl-4-isoxazolepropionic acid (AMPA) receptors, *N*-methyl D-aspartate (NMDA) receptors, and γ-aminobutyric acid, type A (GABA_A_) receptors, while recording from the cultures on MEAs to observe changes in spontaneous activity. The blockers used included the AMPA receptor antagonist 6-cyano-7-nitroquinoxaline-2,3-dione disodium salt (CNQX), the NMDA receptor antagonist D-(-)-2-amino-5-phosphonopentanoic acid (AP5), and the GABA_A_ receptor antagonist bicuculline. Washing in the AMPA_R_ antagonist CNQX on cultures of spiking HB9^+^ neurons caused a gradual decrease in activity to about 40% of initial levels (Figures 5A,E). There was a significant relationship between drug dose and spike rates (linear mixed effects model: β = −0.04, *p* = 2.65e-63). Similarly, CNQX application resulted in a significant decrease in the activity of Chx10^+^ neurons to about 40% of the initial rate (Figures 5B,F) (β = −0.021, *p* = 8.61e-15). The application of CNQX to cocultures caused the majority of cells to abruptly stop bursting (Figure 5C). Other neurons gradually became decoupled from the network bursts and fired tonically for a brief period before also being silenced during CNQX application (Figure 5D). The average response of cocultured neurons to CNQX application reflects this transient increase in activity followed by eventual inhibition (Figure 5G) (β = −0.012, *p* = 0.0015).

We repeated the CNQX drug application on cocultures and used calcium imaging with Rhodamine3 to visualize the activity of the culture prior to and after application of 40 μM CNQX. Despite a loss of network bursting activity, we observed that some HB9^+^ neurons in the coculture continued to have spontaneous spiking activity in the presence of a blocking concentration of CNQX (Figures 5H–J and Supplementary Videos S7–S9).

**FIGURE 6:**
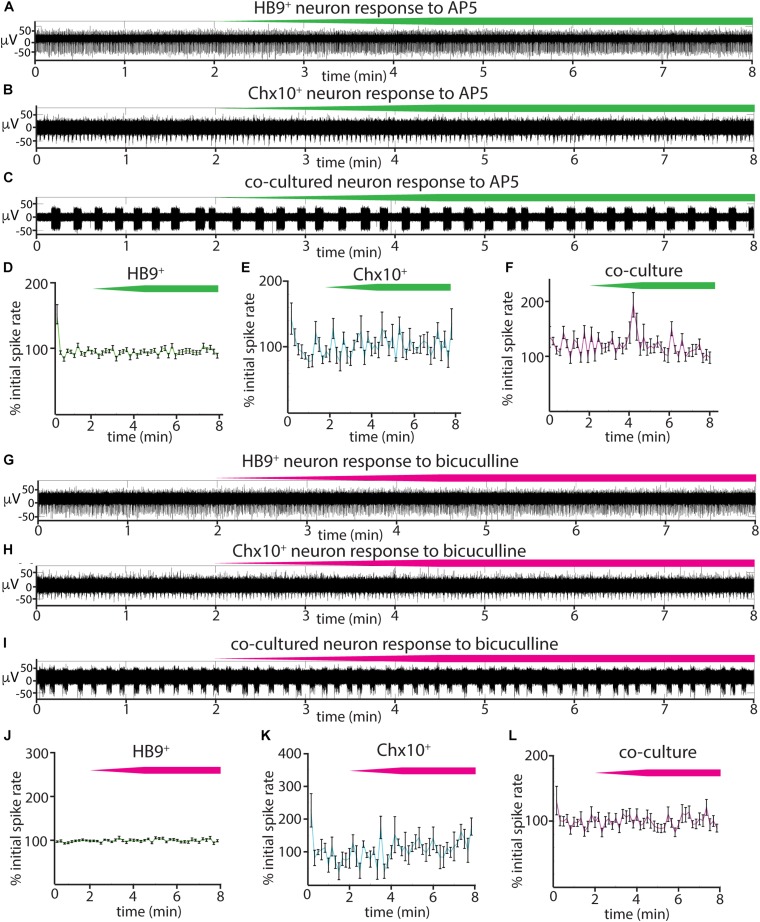
Responses of reticulospinal cultures to NMDA_R_ and GABA_A_R blockers. **(A–C)** Examples of high pass filtered MEA recordings of spiking neurons during wash-in of a 500 μM solution of NMDA_R_ blocker AP5 at 50 μL/min (final AP5 concentration 50 μM), green bars show approximate time course of AP5 wash-in. **(A)** Neuron from HB9:GFP^+^ culture, **(B)** neuron from Chx10:CFP^+^ culture, **(C)** neuron from HB9:GFP^+^/Chx10:CFP^+^ coculture. **(D–F)** Normalized mean responses of all recorded neurons to AP5 wash-in, **(D)** HB9:GFP^+^ cultures (*n* = 3), **(E)** Chx10:CFP^+^ cultures (*n* = 3), **(F)** HB9:GFP^+^/Chx10:CFP^+^ cocultures (*n* = 4). **(G–I)** Examples of high pass filtered MEA recordings of spiking neurons during wash-in of a 600 μM solution of GABA_A_R blocker bicuculline at 50uL/min (final bicuculline concentration 60 μM). Magenta bars show time course of bicuculline wash-in. **(G)** Neuron from HB9:GFP^+^ culture, **(H)** neuron from Chx10:CFP^+^ culture, **(I)** neuron from HB9:GFP^+^/Chx10:CFP^+^ coculture. **(J–L)** Normalized mean responses of all recorded neurons to bicuculline wash-in, **(J)** HB9:GFP^+^ cultures (*n* = 3), **(K)** Chx10:CFP^+^ cultures (*n* = 3), **(L)** HB9:GFP^+^/Chx10:CFP^+^ cocultures (*n* = 4).

We also tested the effects of the NMDA receptor antagonist AP5 on all three cultures (Figures 6A–F) and found that there was no significant relationship between blocker dose and spike rates during AP5 wash-in (linear mixed effects model for: HB9^+^ neurons, β = 0.0005 *p* = 0.23, Chx10^+^ neurons, β = 0.004, *p* = 0.25, coculture, β = 0.006, *p* = 0.24). The GABA_A_ receptor blocker bicuculline also had no detectable effect on Chx10^+^ hindbrain neurons, HB9^+^ motor neurons, or cocultures (Figures 6G–L) (linear mixed effects model for: HB9^+^ neurons, β = 0.0003, *p* = 0.54, Chx10^+^ neurons, β = 0.0026, *p* = 0.34, coculture, β = 0.0057, *p* = 0.13).

## Discussion

In this study, we used flow cytometry to isolate HB9^+^ motor neurons and Chx10^+^ hindbrain neurons and cultured these cell types separately and together to form a reticulospinal circuit. We found that the sorting process did not significantly impact the development of HB9^+^ and Chx10^+^ neuron electrophysiology. When isolated, these two cell types developed distinct patterns of network activity. HB9^+^ neurons tended toward uncoordinated spike trains, while Chx10^+^ hindbrain neurons were characterized by regular, network-wide bursts of activity. Cocultures of these two cell types developed the network bursts characteristic of Chx10^+^ neurons that recruited neighboring HB9^+^ neurons. We further note that the activity of all these cultures was insensitive to NMDA and GABA_A_ receptor blockers but could be inhibited by the AMPA receptor blocker CNQX.

### Effect of Cell Sorting on Electrophysiology of Isolated Cells

Although HB9 plays an important role in consolidating motor neuron identity (Arber et al., 1999; Thaler et al., 1999), we note that there exists a second population of HB9^+^ interneurons with a rhythm-generating role in the spinal cord (Hinckley et al., 2005; Wilson et al., 2005, 2007; Caldeira et al., 2017). These interneurons are distinguished from cholinergic motor neurons by their lack of ChAT immunoreactivity. Other studies have found that over 95% of the HB9^+^ neurons derived from HB9:GFP stem cells using the protocol developed by Wichterle et al. (2002) are ChAT^+^, indicating that HB9^+^ ChAT^–^ interneurons, although prominent in the spinal cord, form a minor segment of the total HB9^+^ population of stem cell derived neurons (Wichterle et al., 2002; Miles et al., 2004; Di Giorgio et al., 2007; López-González et al., 2009). Therefore, we consider our FACS-sorted stem cell-derived HB9^+^ neurons to primarily have a motor neuron identity.

FACS-sorted stem-cell derived HB9^+^ motor neurons develop complex morphology and electrical excitability *in vitro* (Wichterle et al., 2002; Haidet-Phillips et al., 2011; Yang et al., 2013; Uzel et al., 2016), but previous studies had not established whether the nature of their electrical responses had been altered. Our results show that sorted Hb9^+^ motor neurons develop spontaneous spiking activity, fast inactivating sodium currents, and repetitive trains of action potentials in response to current injection. These results are consistent with the reported electrophysiology of unsorted stem cell-derived motor neurons (Miles et al., 2004). Thus, our single-cell electrophysiology indicates that the presence of other neuronal subtypes and progenitors does not alter the electrical properties of HB9^+^ motor neurons, indicating that they are determined by cell type identity.

Hindbrain Chx10^+^ neurons comprise a very small subset of the total hindbrain (Figure 1D), which confounds attempts to study their network activity in unsorted cultures. We found that FACS-sorted Chx10^+^ hindbrain neurons developed spontaneous rhythmic bursting activity *in vitro*. This behavior is consistent with the observation that a closely related population of spinal V2a neurons develops spontaneous rhythmic activity following FACS isolation and reaggregation into three-dimensional cultures (Sternfeld et al., 2017). The resting membrane potential of the hindbrain Chx10^+^ neurons started off with a resting potential close to 0 mV, but became increasingly negative as they matured and developed spontaneous activity. Immature neurons tend to have a depolarized resting membrane potential that becomes increasingly hyperpolarized with age (Tyzio et al., 2003). This well documented observation is thought to arise from a combination of real developmental changes in ionic conductances (Spigelman et al., 1992; Martin-Caraballo and Greer, 1999; Ben-Ari, 2002) and leak current artifacts introduced by the patch clamp pipette that are particularly strong in immature neurons, which have a high input resistance (Tyzio et al., 2003).

In this study, we selectively isolated and cultured Chx10^+^ neurons of the E12.5 embryonic hindbrain, which specifically excludes spinal Chx10^+^ V2a neurons. However, we note that even this spatially and molecularly defined population of neurons is likely to contain a considerable degree of diversity, as demonstrated by the different roles that hindbrain Chx10^+^ neurons play in regulating respiratory rhythms (Crone et al., 2012) and hindlimb locomotion (Bretzner and Brownstone, 2013; Bouvier et al., 2015).

Taken together, these experiments demonstrate that FACS is a viable option for the isolation and subsequent long-term culture of molecularly defined neuronal subtypes.

### Cell Type Specific Patterns of Activity in Cultures of Sorted Neurons

Several prior studies have arranged neurons on MEAs in very specific patterns (Maher et al., 1999; Wheeler and Brewer, 2010), but not defined subtypes. The random patterning of molecularly defined cells on our arrays allowed us to explore whether there is a consistent influence of cell type on network behavior, regardless of network architecture.

Our observation that HB9^+^ motor neurons fail to develop spontaneous activity in the absence of glia is consistent with other studies that have demonstrated the essential support that astrocytes provide for cultured neurons (Wang et al., 1994; Boehler et al., 2007), including motor neurons (Ullian et al., 2004). When we cultured sorted HB9^+^ neurons with astrocytes they developed unsynchronized spike trains. FACS appears to be critical for this behavior, as previous studies of stem cell-derived HB9^+^ motor neurons cultured without FACS isolation reported coordinated network bursts (Jenkinson et al., 2017). In unsorted cultures, a cell type other than HB9^+^ neurons must have contributed to the generation of this activity pattern. We note that the spinal motor neuron differentiation protocol generates a small but prominent subpopulation of spinal V2a neurons, a cell type that is closely related to our rhythmogenic hindbrain Chx10^+^ neurons (Brown et al., 2014).

We found that Chx10^+^ hindbrain neurons isolated by FACS and cultured on MEAs developed robust and highly coordinated network bursts. Calcium imaging (Figure 3K) indicates that virtually all Chx10^+^ neurons participate in these bursts, with no discernible time delay. Thus, simultaneous spiking is an intrinsic feature of Chx10^+^ neurons in culture and does not appear to require the presence of other neuron types.

### Chx10-Like Pattern of Activity Is Dominant in Coculture

Recordings from the coculture indicate that HB9^+^ neurons develop rhythmic bursting activity that is correlated with that of neighboring Chx10^+^ neurons (Figure 4E). The HB9^+^ neurons’ activity under these conditions noticeably differed from their behavior in monoculture, where they failed to develop coordinated network activity. In contrast, Chx10^+^ hindbrain neurons were able to generate their own patterns of activity in monoculture without the need for exogenous cell types besides astrocytes. Taken together, these results suggest that in coculture, Chx10^+^ neurons are driving patterned HB9^+^ neuron activity, although it is as yet unclear whether this interaction is the result of direct innervation of HB9^+^ neurons by Chx10^+^ neurons or some indirect effect that Chx10^+^ neurons exert via diffusible factors or changes to HB9^+^ neurons’ intrinsic excitability.

Our results indicate that electrically excitable cell types develop different spontaneous patterns of activity that are driven by the intrinsic properties of that cell type. However, the genetic identity of the neurons being cultured is not the sole determinant of their network behavior. Astrocytes play an important role in modulating neuronal activity, as we were unable to detect any spontaneous activity in cultures lacking astrocytes, consistent with previous results with neuronal culture on MEAs (Ullian et al., 2004; Boehler et al., 2007). It is likely that one way that astrocytes facilitate neuronal activity is by removing excess glutamate to prevent excitotoxicity (Rothstein et al., 1996; Swanson et al., 1997). Consistent with previous reports (Scemes and Giaume, 2006), the astrocytes in our culture were active, as indicated by slow waves of calcium activity which we were able to observe in calcium imaging (Supplementary Video S1), but which did not produce electrical excitation on MEAs.

Our observation that Chx10^+^ neurons are able to impose temporally patterned activity on HB9^+^ neurons is consistent with their *in vivo* function of driving rhythmic behaviors such as hindlimb locomotion and respiration. Prior studies suggest that activation of these neurons is associated with bouts of locomotion, and may drive locomotor stop and turn signals (Bretzner and Brownstone, 2013; Bouvier et al., 2015; Cregg et al., 2019). Additionally, Chx10^+^ neurons project to the pre-Bötzinger complex, and their ablation disrupts respiratory rhythms in newborn mice, with normal respiratory rhythms gradually reasserting themselves as the mice grow older (Crone et al., 2009, 2012).

### Emergent Properties of Neuronal Cultures as Revealed by Synaptic Inhibition

Our results from applying a panel of synaptic blockers targeting AMPA, NMDA, and GABA_A_ receptors to spontaneously active HB9^+^ and Chx10^+^ neuron cultures (Figures 5, 6) show that the AMPA_R_ blocker CNQX effectively blocked all bursts in Chx10^+^ cultures and significantly decreased the activity in HB9^+^ neuron cultures. This is consistent with the observation that spinal motor neurons cultured *in vitro* form glutamatergic synapses that are entirely blocked by CNQX (Ullian et al., 2004). CNQX application similarly eradicates spontaneous network bursting in cultures of spinal Chx10^+^ neurons that are otherwise insensitive to glycine and GABA antagonists (Sternfeld et al., 2017). Our finding that bursts of hindbrain Chx10^+^ neurons could be effectively eradicated by blocking glutamatergic transmission suggests that the robust rhythmicity of these neurons is an emergent property of the network, as opposed to pacemaker activity generated by individual cells. This contrasts with true pacemaker neurons, such as those of the pre-Bötzinger complex, where bursts are intrinsic to individual cells, and therefore insensitive to the same cocktail of synaptic blockers (Chevalier et al., 2016). Thus, we observe that AMPA receptor activation can drive very different outcomes that depend on cell type.

When we applied CNQX to the coculture, some neurons switched from rhythmic bursting to a transient period of tonic spiking before becoming quiescent. This emergent property may be driven by HB9^+^ neurons that revert to their native spiking phenotype in the absence of the patterning influence of network bursts. This is consistent with our calcium imaging data in which we identified HB9^+^ neurons in coculture that continued to have calcium spikes even in the presence of a dose of CNQX that effectively disrupted network bursts (Figure 5I). CNQX is also known to act as a partial AMPA receptor agonist under certain conditions, which could explain why some neurons in the coculture condition increased their firing rate following CNQX application (Menuz et al., 2007).

### Implications of Our Results for Modeling Reticulospinal Circuits

The results of our study can be applied to modeling of reticulospinal circuits, different aspects of which are currently being examined by multiple groups (Pivetta et al., 2014; Sternfeld et al., 2017; Oueghlani et al., 2018). However, in the rodent reticulospinal circuit, hindbrain Chx10^+^ neurons primarily contact premotor networks within the spinal cord, as opposed to synapsing directly onto motor neurons the way that we have modeled in our cultures (Bouvier et al., 2015; Cregg et al., 2019). Thus, further elaboration of our Chx10-HB9 coculture model is required to fully recapitulate the mammalian reticulospinal circuit. In the mammalian motor system, reticulospinal projections provide descending input to spinal central pattern generators that is then translated into the patterned output onto motor neurons essential for the appropriate expression of gait (Grillner et al., 2008). This reticulospinal signal, although important for initiating and halting locomotion, is broadly unpatterned (Juvin et al., 2016; Capelli et al., 2017; Grätsch et al., 2019). So, incorporating additional spinal interneuron cell types that participate in the premotor central pattern generator would provide an essential layer of complexity to our reticulospinal culture that could potentially model how descending signals from glutamatergic hindbrain neurons like the Chx10^+^ population are transformed into rhythmic locomotor-like activity in an experimentally tractable system.

Despite such caveats, it can be argued that the circuit created by our *in vitro* cocultures is similar to the basic circuitry found in fish and amphibians. In the zebrafish hindbrain Chx10^+^ neurons directly contact spinal motor neurons and generate swimming when selectively stimulated (Kimura et al., 2013). Likewise, in *Xenopus* tadpoles the Chx10^+^ dorsoventral hindbrain provides patterned excitatory input directly to motor neurons, driving sensory-evoked swimming before other motor control systems have developed (Soffe et al., 2009; Li and Soffe, 2019). Thus, even in our highly simplified system, we have been able to recreate some biologically relevant behaviors.

Ultimately, the most generalizable aspect our findings is the observation that the aggregate activity of neuronal networks is influenced by the specific molecular identity of their constituent neurons, beyond specific pacemaker cells or broad categories of excitatory-inhibitory cells. The method that we have developed for isolating specific cell types and culturing them *in vitro* allows us to distinguish the extent to which the properties of a given neuronal class are explained by unique features of its cell type rather than its connectivity within a larger circuit. These aspects would be difficult to parse in an *in vivo* system. Our results demonstrate how certain electrical properties of neurons are intrinsic to their specific subtype, which is an important consideration for modeling the effects of mutations and disease on network function. The cell type compositions of circuit models can have profound effects on patterns of activity and therefore need to be considered and interpreted carefully.

## Data Availability Statement

Calcium imaging datasets generated for this study are included in the manuscript/Supplementary Files. Other raw data, supporting the conclusions of this manuscript will be made available by the authors, without undue reservation, to any qualified researcher.

## Ethics Statement

The animal study was reviewed and approved by The Rockefeller University Animal Care and Use Committee.

## Author Contributions

AB, IT, LK, and DP designed the research. AB and HK performed the experiments. AB and IT wrote the manuscript.

## Conflict of Interest

The authors declare that the research was conducted in the absence of any commercial or financial relationships that could be construed as a potential conflict of interest.

## Supplementary Material

VIDEO S1 Calcium imaging of glia. Time course of Rhodamine3 fluorescence of astrocyte culture with spontaneous calcium activity. Playback of time 1–130 s from raw recording (total time of raw recording is 130 s). Minimum value of each pixel from the entire recording was subtracted from that corresponding pixel in each frame to minimize noise.

VIDEO S2 Calcium imaging of spiking HB9^+^ neurons. Time course of Rhodamine3 fluorescence that corresponds to quantification in Figure 3E. Playback of time 1–12 s from raw recording (total time of raw recording is 80 s). ROIs used to quantify HB9^+^ neuron calcium activity are outlined in yellow. Minimum value of each pixel from the entire recording was subtracted from that corresponding pixel in each frame to minimize noise.

VIDEO S3 Calcium imaging of HB9^+^ neurons with coordinated spiking. Time course of Rhodamine3 fluorescence that corresponds to quantification in Figure 3F. Playback of time 1–15 s from raw recording (total time of raw recording is 80 s). ROIs used to quantify GFP^+^ neuron calcium activity are outlined in yellow. Minimum value of each pixel from the entire recording was subtracted from that corresponding pixel in each frame to minimize noise.

VIDEO S4 Calcium imaging of Chx10^+^ neuron network bursts. Time course of Rhodamine3 fluorescence that corresponds to quantification in Figure 3K. Playback of time 1–130 s from raw recording. ROIs used to quantify Chx10^+^ neuron calcium activity are outlined in yellow.

VIDEO S5 Calcium imaging of HB9^+^/Chx10^+^ neuron coculture. Time course of Rhodamine3 fluorescence that corresponds to quantification in Figure 4E. Playback of time 40–80 s from raw recording (total time of raw recording is 80 s). ROI used to quantify HB9^+^ neuron’s calcium activity is outlined in green, ROI used to quantify Chx10^+^ neuron’s calcium activity is outlined in cyan. Minimum value of each pixel from the entire recording was subtracted from that corresponding pixel in each frame to minimize noise.

VIDEO S6 Calcium imaging of HB9^+^ neurons in HB9^+^/Chx10^+^ neuron coculture. Time course of Rhodamine3 fluorescence that corresponds to quantification in Figure 4F. Playback of time 1–80 s from raw recording. ROIs used to quantify HB9^+^ neuron calcium activity are outlined in green. Minimum value of each pixel from the entire recording was subtracted from that corresponding pixel in each frame to minimize noise.

VIDEO S7 Calcium imaging of bursting HB9^+^/Chx10^+^ neuron coculture prior to AMPA_R_ blocker application. Time course of Rhodamine3 fluorescence that corresponds to quantification in Figure 5H. Playback of time 35–75 s from raw recording (total time of raw recording is 80 s). ROIs used to quantify HB9^+^ neuron calcium activity are outlined in green, ROI used to quantify Chx10^+^ neuron’s calcium activity is outlined in cyan. Minimum value of each pixel from the entire recording was subtracted from that corresponding pixel in each frame to minimize noise.

VIDEO S8 Calcium imaging of HB9^+^ neurons in co-culture after application of 40 μM AMPA_R_ blocker. Time course of Rhodamine3 fluorescence that corresponds to quantification in Figure 5I. Playback of time 8–38 s from raw recording (total time of raw recording is 80 s). ROIs used to quantify HB9^+^ neuron calcium activity are outlined in green, ROI used to quantify Chx10^+^ neuron’s calcium activity is outlined in cyan. Minimum value of each pixel from the entire recording was subtracted from that corresponding pixel in each frame to minimize noise.

VIDEO S9 Calcium imaging of bursting HB9^+^/Chx10^+^ neuron coculture after washout of AMPA_R_ blocker. Time course of Rhodamine3 fluorescence that corresponds to quantification in Figure 5J. Playback of time 10–40 s from raw recording (total time of raw recording is 80 s). ROIs used to quantify HB9^+^ neuron calcium activity are outlined in green, ROI used to quantify Chx10^+^ neuron’s calcium activity is outlined in cyan. Minimum value of each pixel from the entire recording was subtracted from that corresponding pixel in each frame to minimize noise.
